# FlyNap (Triethylamine) Increases the Heart Rate of Mosquitoes and Eliminates the Cardioacceleratory Effect of the Neuropeptide CCAP

**DOI:** 10.1371/journal.pone.0070414

**Published:** 2013-07-16

**Authors:** Weihan Chen, Julián F. Hillyer

**Affiliations:** Department of Biological Sciences, Vanderbilt University, Nashville, Tennessee, United States of America; Centro de Pesquisas René Rachou, Brazil

## Abstract

FlyNap (triethylamine) is commonly used to anesthetize *Drosophila melanogaster* fruit flies. The purpose of this study was to determine whether triethylamine is a suitable anesthetic agent for research into circulatory physiology and immune competence in the mosquito, *Anopheles gambiae* (Diptera: Culicidae). Recovery experiments showed that mosquitoes awaken from traditional cold anesthesia in less than 7 minutes, but that recovery from FlyNap anesthesia does not begin for several hours. Relative to cold anesthesia, moderate exposures to FlyNap induce an increase in the heart rate, a decrease in the percentage of the time the heart contracts in the anterograde direction, and a decrease in the frequency of heartbeat directional reversals. Experiments employing various combinations of cold and FlyNap anesthesia then showed that cold exposure does not affect basal heart physiology, and that the differences seen between the cold and the FlyNap groups are due to a FlyNap-induced alteration of heart physiology. Furthermore, exposure to FlyNap eliminated the cardioacceleratory effect of crustacean cardioactive peptide (CCAP), and reduced a mosquito’s ability to survive a bacterial infection. Together, these data show that FlyNap is not a suitable substitute to cold anesthesia in experiments assessing mosquito heart function or immune competence. Moreover, these data also illustrate the intricate biology of the insect heart. Specifically, they confirm that the neurohormone CCAP modulates heart rhythms and that it serves as an anterograde pacemaker.

## Introduction

A common need in experiments evaluating biological processes in mosquitoes is the complete immobilization of the insect. For example, mosquito anesthesia is needed to record certain physiological process, to collect tissue samples, and to perform organismal manipulations such as injections. The gold standard for mosquito immobilization is cold-induced anesthesia [1–3], although mosquitoes are also frequently anesthetized by exposure to CO_2_ [4,5]. Both of these methods, while effective, have their limitations. Mainly, cold- and CO_2_-induced anesthesia require continuous exposure to low temperatures and CO_2_, respectively, and prolonged subjection to these conditions can be lethal to the insect. To our knowledge, mosquito anesthesia via exposure to chemical vapors has not been reported, with the exception of several lab and field studies that used lethal doses of triethylamine to permanently immobilize (and kill) mosquitoes [6–12]. However, triethylamine, often used as a commercial preparation called FlyNap, has become a commonplace tool for *Drosophila* sp. experiments in both research and teaching settings, and has been shown to immobilize fruit flies for up to several hours [13–16]. The purpose of this study was to determine whether FlyNap (triethylamine) is a suitable anesthetic agent for mosquito research.

Prolonged mosquito immobilization is required for the measurement of circulatory physiology. In the standard procedure, a mosquito is cold-anesthetized, pins are placed against the body and through non-vascular areas of the wings, and intravital video imaging of the contracting heart or another area of the insect is then performed [17,18]. Using this methodology it has been shown that (1) hemolymph circulation is primarily driven by a dorsal vessel that extends the length of the insect and is subdivided into a thoracic aorta and an abdominal heart; (2) wave-like contractions of the heart periodically alternate between propelling hemolymph in the anterograde (toward the head) and retrograde (toward the posterior abdomen) directions; (3) hemolymph enters the dorsal vessel through incurrent ostia located in each abdominal segment and exits through excurrent openings located at the anterior and posterior ends of the insect; (4) abdominal contractions propel extracardiac hemolymph; and (5) certain neuropeptides modulate heart contraction dynamics [17–20]. Some of the experiments that led to the above conclusions required that mosquitoes be physically immobilized for periods longer than 1 h. Because mosquitoes fully recover from cold or CO_2_ anesthesia in less than 10 min, the identification of anesthetic agents that knock down mosquitoes for prolonged periods of time would facilitate studies into circulatory physiology.

Anesthesia is also commonly required in studies assessing mosquito immune competence. For example, adult mosquitoes must be anesthetized prior to (1) injecting pathogens into the hemocoel; (2) injecting double stranded RNA for gene knockdown; (3) injecting chemicals that inhibit immune pathways; (4) performing intravital video imaging; and (5) collecting specific tissues [1,2,5,21–26]. The above approaches have led to discoveries on the molecular bases of immune responses, the role immune cells play in combating infections, the ability of pathogens to complete their life cycles in mosquitoes, and the ability of mosquitoes to overcome infections [27–29]. Cold or CO_2_ anesthesia is well suited for many of these studies because most of the procedures employed are of short duration. Nevertheless, the identification of a long lasting anesthetic agent would be immensely beneficial for experiments that require intravital video imaging.

In this study we compared the effect of cold-induced anesthesia and FlyNap-induced anesthesia on mosquito heart contraction dynamics and immune competence. We found that exposure to FlyNap moderately elevates heart rates, biases the proportional directionality of heart contractions, eliminates the heart’s response to the cardioacceleratory neuropeptide CCAP, and decreases the ability of mosquitoes to survive the early stages of a bacterial infection.

## Materials and Methods

### Mosquito rearing

*Anopheles gambiae* (Diptera: Culicidae), G3 strain, were reared in an environmental chamber at 27 °C and 75% relative humidity as described [18]. Adult mosquitoes were fed a 10% sucrose solution ad libitum, and all experiments were carried out on adult females at 4 days post-eclosion.

### Mosquito anesthesia

Mosquitoes were anesthetized using temperature- or chemical-based methods. For cold anesthesia, mosquitoes were exposed to -20 °C for 60 sec, and were then placed on a cold petri dish until use. This procedure is customary for studies assessing mosquito circulation and immunity [18,25]. Chemical anesthesia was performed by placing mosquitoes in a covered 60 cm^3^ enclosure (100 ml beaker) and inserting into the enclosure an absorbent wand that had been loaded with a triethylamine-based cocktail called FlyNap (Carolina Biological Supply, Burlington, NC). The absorbent wand was loaded with FlyNap by immersion, and excess solution was removed from the wand by rolling it on a paper towel. This resulted in 19 µl +/- 3 µl S.D. of FlyNap being loaded onto the wand. Following exposure to FlyNap, mosquitoes were placed on a petri dish at room temperature until use. Mosquito enclosures were thoroughly washed prior to being reused. Mosquito anesthesia by FlyNap occurs through exposure to triethylamine vapors, with the composition of FlyNap being 50% triethylamine (active ingredient), 25% ethanol and 25% fragrances.

### Mosquito recovery from anesthesia

Following cold or FlyNap (10 sec, 30 sec, 1 min or 5 min exposure) treatment, mosquitoes were transferred to 473 cm^3^ containers with a fine mesh marquisette top. Recovery from anesthesia was recorded every minute for 15 min and every hour for 12 h, with mosquitoes marked as recovered when they could stand and fly. Then, in a similar manner, mosquito survival was tracked for 7 days. Four independent trials were conducted (except for 10 sec FlyNap, which was only included in 2 of the trials), with each trial being composed of 15 mosquitoes per group.

### Measurement of mosquito heart physiology

To measure heart contraction dynamics, mosquitoes were restrained dorsal side up on Sylgard elastomer plates as previously described and pictured [17]. Briefly, mosquitoes were restrained by placing pins (1) against (not through) the pronotal lobes of the anterior mesothorax (2), through non-vascular areas of the wings, and (3) against (not through) the lateral abdomen. Contracting mosquito hearts were then visualized through the dorsal abdominal cuticle using bright field epi and trans illumination on a Nikon SMZ1500 stereomicroscope (Tokyo, Japan) connected to a Photometrics, Cool Snap HQ2 CCD camera (Roper Scientific, Ottobrunn, Germany) and Nikon NIS-Elements Advanced Research software. To calculate heart contraction dynamics, 60 sec videos of the entire abdomen of each mosquito were acquired and the following parameters were manually measured: (1) the total contraction rate (in beats per second; Hertz; Hz); (2) the anterograde contraction rate (rate when contractions propagate toward the head); (3) the retrograde contraction rate (rate when contractions propagate toward the posterior abdomen); (4) the frequency of heartbeat directional reversals (frequency of switches from anterograde to retrograde and vice versa); (5) the percent time the heart spent contracting in the anterograde and retrograde directions; and (6) the length of anterograde and retrograde contraction periods (amount of time the heart spent contracting in a given direction prior to a heartbeat directional reversal). For the measurement of this last parameter only the data from complete contraction periods was used. That is, for each video, data from the first and last contraction periods were discarded as the start of the first period and the end of the last period were not contained within the recording.

Regardless of the method of anesthesia, all mosquito recordings were performed after the body had normalized to room temperature (~22 °C), and unless stated otherwise, readings were acquired at 1-5 min post-treatment. Also, when multiple heart recordings of a single mosquito were acquired, the mosquito remained restrained for the entirety of the experiment. Statistical analysis of data comparing cold exposure to exposure to FlyNap (10 sec, 30 sec, 1 min or 5 min), or the effect of multiple combinations of anesthesia, was performed by one-way ANOVA. When a statistical difference was found (P<0.05), Tukey’s multiple comparisons post-hoc test was performed to compare cold anesthesia (treated as the reference group, or R) to the other treatment groups. For the experiment testing how heart physiology changes between 1 min and 1 h following 30 sec FlyNap treatment, data were statistically analyzed using the paired two-tailed t-test. For videos of contracting hearts imaged using this methodology, see our previously published work [17–19].

### Treatment with CCAP

Mosquitoes that had been anesthetized via exposure to cold or via exposure to FlyNap for 30 sec were restrained and a 60 sec video recording of the contracting heart was acquired as above (basal heart levels). An approximate volume of 0.1 µl of either PBS or 1 X 10^-5^ M CCAP in PBS was then intrathoracically injected into each restrained mosquito. These injections were performed using a finely pulled capillary glass needle that was inserted into the mosquito through the anepisternal cleft of the lateral mesothorax. After allowing the injected solution to disseminate throughout the hemocoel for 10 min, a second 60 sec video recording was acquired (heart levels after injection) and heart physiology was measured as above. Data from this experiment, which are paired data (contraction dynamics before and after the injection), were initially statistically analyzed using repeated-measures two-way ANOVA. Sidak’s multiple comparisons post-hoc test was then performed to detect statistical differences within each group (pre- versus post-injection).

### Mosquito survival following infection

Mosquitoes were anesthetized by exposure to cold or by exposure to FlyNap for 30 sec. For each method of anesthesia, one subset of mosquitoes was intrathoracically injected with Luria Bertani’s rich nutrient medium (LB broth; termed the injured group). A second subset was injected with *E. coli* (modified dh5 alpha; termed the infected group) that had been grown overnight in LB broth [23,24], and a third subset was not injected (termed the naïve group). All groups were housed in 473 cm^3^ containers with a fine mesh marquisette top and were fed a 10% sucrose solution *ad libitum*. Mosquito survival was then recorded every day for 14 days. Four independent trials were conducted, with each trial containing 30 female mosquitoes per group (180 mosquitoes per trial). For each trial and treatment, the survival curves of cold exposed and FlyNap exposed mosquitoes were compared using the Logrank test. Finally, the precise *E. coli* infection dose used in each trial was calculated immediately following mosquito injections by plating serial dilutions of the overnight culture on LB agar plates, growing them overnight at 37 °C, and then counting the resultant colony forming units.

## Results

### Recovery from cold and FlyNap anesthesia: minutes versus hours

Exposure to cold anesthesia for 60 sec anesthetized 100% of the mosquitoes, as did exposure to FlyNap for 30 sec, 1 min and 5 min. Exposure to FlyNap for 10 sec resulted in only some of the mosquitoes becoming anesthetized. Those that became anesthetized remained immobilized for several hours, but those that were not could simply fly away. For that reason, the data presented below for 10 sec FlyNap exposure includes only those mosquitoes that became anesthetized by the procedure.

**Figure 1:**
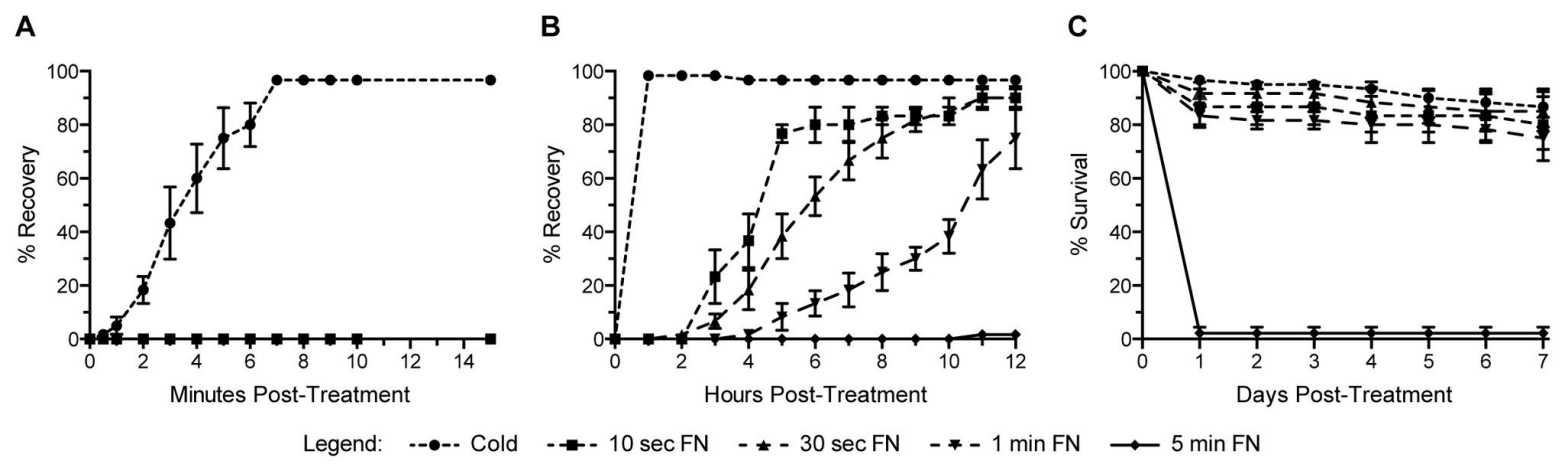
Recovery and survival following cold or FlyNap anesthesia. Mosquitoes were anesthetized by exposure to cold for 60 sec or by exposure to FlyNap (FN) for 10 sec, 30 sec, 1 min or 5 min. (A–B) Percent of mosquitoes that recovered from anesthesia during the first 15 min (A) and 12 h (B) following treatment. (C) Percent of mosquitoes that survived during the first 7 days following anesthesia. Data are the average of 4 trials, each composed of 15 mosquitoes per treatment group.

Following cold anesthesia, 5% of the mosquitoes recovered (could stand and fly) by 1 min after being returned to room temperature, 60% recovered by 4 min, and 100% recovered by 7 min (Figure 1A). By contrast, of all the mosquitoes treated with FlyNap, only 1 out of 180 recovered by 2 h post-treatment. Treatment with FlyNap for 10 sec, 30 sec or 1 min resulted in >20% recovery by 3 h, 5 h and 8 h post-treatment, respectively, and >50% recovery was observed by 5 h, 6 h, and 11 h respectively (Figure 1B). By 12 h post-treatment, 90%, 90%, 75%, and 2% of the mosquitoes treated with FlyNap for 10 sec, 30 sec, 1 min or 5 min had recovered, respectively, and further recovery was minimal. Fewer mosquitoes recovered in the FlyNap groups when compared to the cold group, but if a mosquito survived the first 24 h following anesthesia (any treatment), it was equally likely to survive the next 7 days, as the survival slopes from days 1 through 7 were similar for all treatment groups (Figure 1C). Altogether, these data show that (1) cold anesthetized mosquitoes recover within minutes of exposure whereas FlyNap anesthetized mosquitoes are immobilized for several hours, (2) not all mosquitoes become anesthetized after 10 sec exposure to FlyNap, and (3) 5 min exposure to FlyNap is lethal. Thus, because 30 sec exposure is the shortest dose that resulted in reliable mosquito anesthesia, it was used the dose used for the majority of the experiments assessing heart physiology and immunity.

### Heart physiology following cold or FlyNap anesthesia: basal heart levels

**Figure 2:**
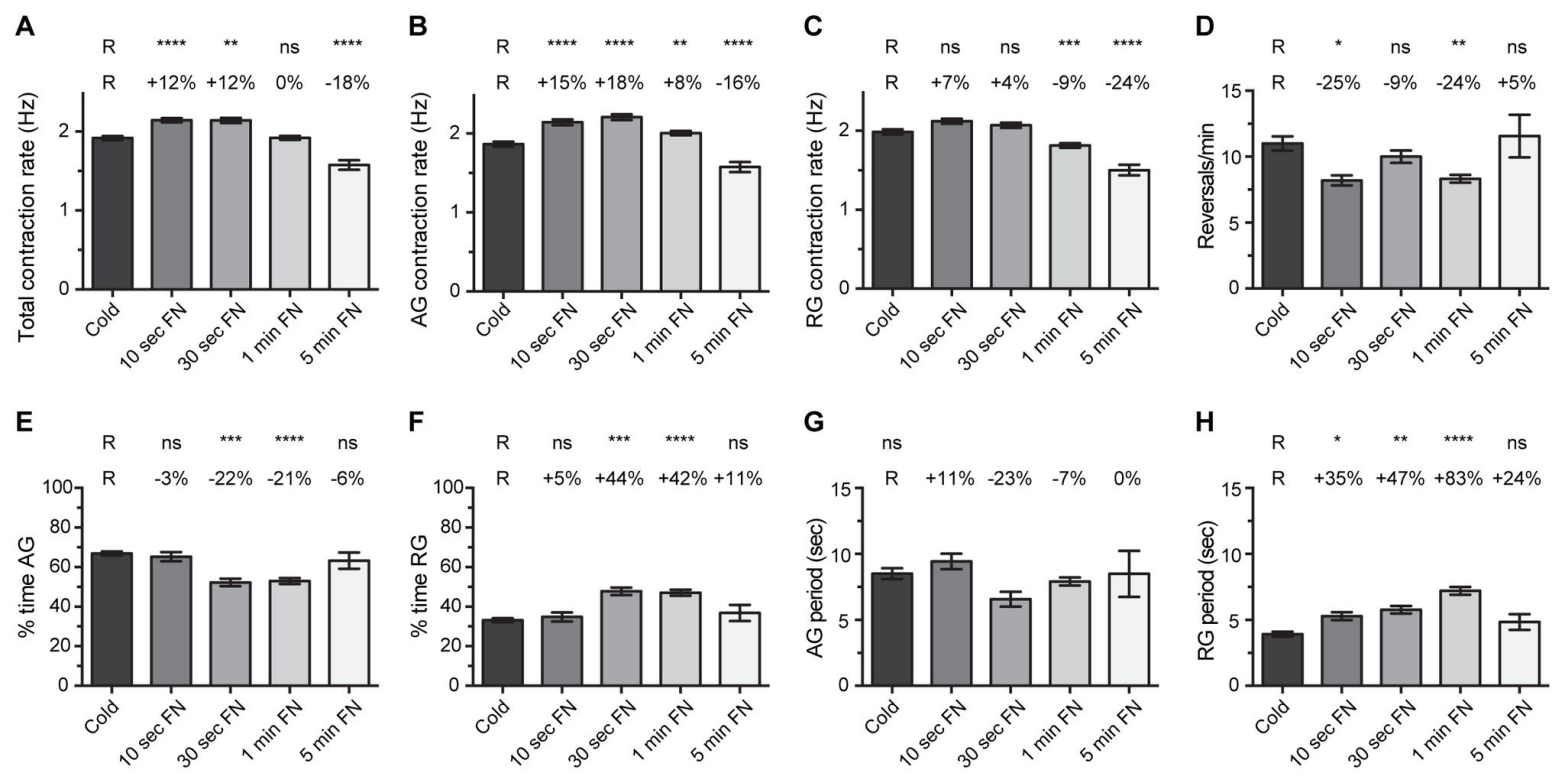
Heart physiology following cold or FlyNap anesthesia. Five groups were assayed: cold anesthesia, and 10 sec, 30 sec, 1 min or 5 min exposure to FlyNap (FN). The following heart parameters are reported: (A–C) total, anterograde and retrograde contraction rate; (D) frequency of heartbeat directional reversals; (E–F) percent time contracting in the anterograde and retrograde directions; (G–H) anterograde and retrograde period lengths. Data were analyzed by one-way ANOVA, and when P<0.05 the statistical significances between the individual FlyNap groups and the cold group (Reference group, R) are denoted using asterisks (Tukey’s multiple comparisons post-hoc test: ns, P>0.05; *, P<0.05; **, P<0.01; ***, P<0.001; ****, P<0.0001). The percentages reported above the columns report the percentage difference between that group and the cold (R) group. Bars denote the standard error of the mean. Sample sizes: Cold, 88; 10 sec FN, 30; 30 sec FN, 20; 1 min FN, 80; 5 min FN, 30.

The heart of cold anesthetized mosquitoes contracted at an average rate of 1.92 Hz (Figure 2A). When separated by contraction direction, the heart contracted at 1.87 Hz during anterograde contraction periods (contractions toward the head) and at 1.98 Hz during retrograde contraction periods (contractions toward the posterior of the abdomen; Figure 2B-C). The heart reversed contraction direction an average of 11 times per minute, with the heart spending 67% of the time contracting in the anterograde direction and 33% of the time contracting in the retrograde direction (Figure 2D–F). Finally, the heart spent an average of 8.5 sec contracting anterograde, and following a heartbeat directional reversal, the heart spent an average of 3.92 sec contracting retrograde (Figure 2G-H). These values are in general agreement with our previous data on basal mosquito heart physiology [18–20].

When compared to cold-anesthesia, heart rates were moderately different following FlyNap exposure, with the magnitude and direction of the difference being dependent on the amount of time mosquitoes were exposed to the triethylamine-based anesthetic agent. Short exposures to FlyNap (10-30 sec) led to higher heart rates when compared to cold anesthesia (12% higher), whereas longer exposures to FlyNap led to either similar heart rates (1 min, no difference) or significantly lower heart rates (5 min, 18% lower; Figure 2A). Treatment (cold and different exposures to FlyNap) did not preferentially affect heart rates in a specific direction: within the treatment groups the average difference between anterograde and retrograde contraction rates was 0.11 Hz, with the differences ranging between 0.07 and 0.19 Hz (Figure 2B-C).

Relative to cold anesthesia, exposure to FlyNap for 30 sec or 1 min led to a decrease in the percentage of time the heart spent contracting in the anterograde direction, and consequently, led to an increase in the amount of time the heart spent contracting in the retrograde direction (Figure 2E-F). As we have previously reported and show again here, the heart of cold-anesthetized mosquitoes spends approximately two-thirds of the time contracting in the anterograde direction and 1/3 of the time contracting in the retrograde direction [18–20]. Mosquitoes exposed to FlyNap for 10 sec or 5 min retained this 2:1 ratio, but exposure to FlyNap for either 30 sec or 1 min led to a 21-22% decrease in the amount of time the heart spent contracting in the anterograde direction, and consequently, these mosquitoes experienced a 42-44% increase in the percentage of time the heart spent contracting in the retrograde direction.

Throughout our studies into mosquito cardiac physiology, the heart parameter that has consistently exhibited the highest amount of variance is the frequency of heartbeat directional reversals [18–20]. In the present study, while this parameter again exhibited a large amount of variance, the general trend was that short or moderate exposures to FlyNap (10 sec to 1 min) led to a decrease in the frequency of heartbeat directional reversals (Figure 2D). When the frequency of reversals decreases, the length (time) of directional contraction periods increases, which in this case resulted primarily in an increase in the number of consecutive seconds the heart spent contracting retrograde before experiencing a heartbeat directional reversal (Figure 2G-H).

Together, these data show that, relative to cold anesthesia, low or moderate (10-30 sec) exposures to FlyNap result in increased heart rates and a change in the proportional directionality of heart contractions. As the FlyNap exposure is increased and moves into the range that is ultimately lethal (5 min), heart rates decrease as physiological processes are permanently disrupted.

### Heart physiology following cold and FlyNap anesthesia: effect of combining the methods

A main finding in the above experiment is that exposure to FlyNap for <1 min leads to heart rates that are elevated when compared to exposure to cold. In an attempt to determine whether FlyNap elevates heart rates or whether the temporary lowering of body temperature during cold anesthesia decreases heart rates, mosquitoes were subject to anesthesia in various combinations. Specifically, mosquitoes were either (1) exposed to cold, (2) exposed to FlyNap for 30 sec; (3) exposed to FlyNap for 30 sec and this was immediately followed by exposure to cold; or (4) exposed to cold and this was immediately followed by exposure to FlyNap for 30 sec.

**Figure 3:**
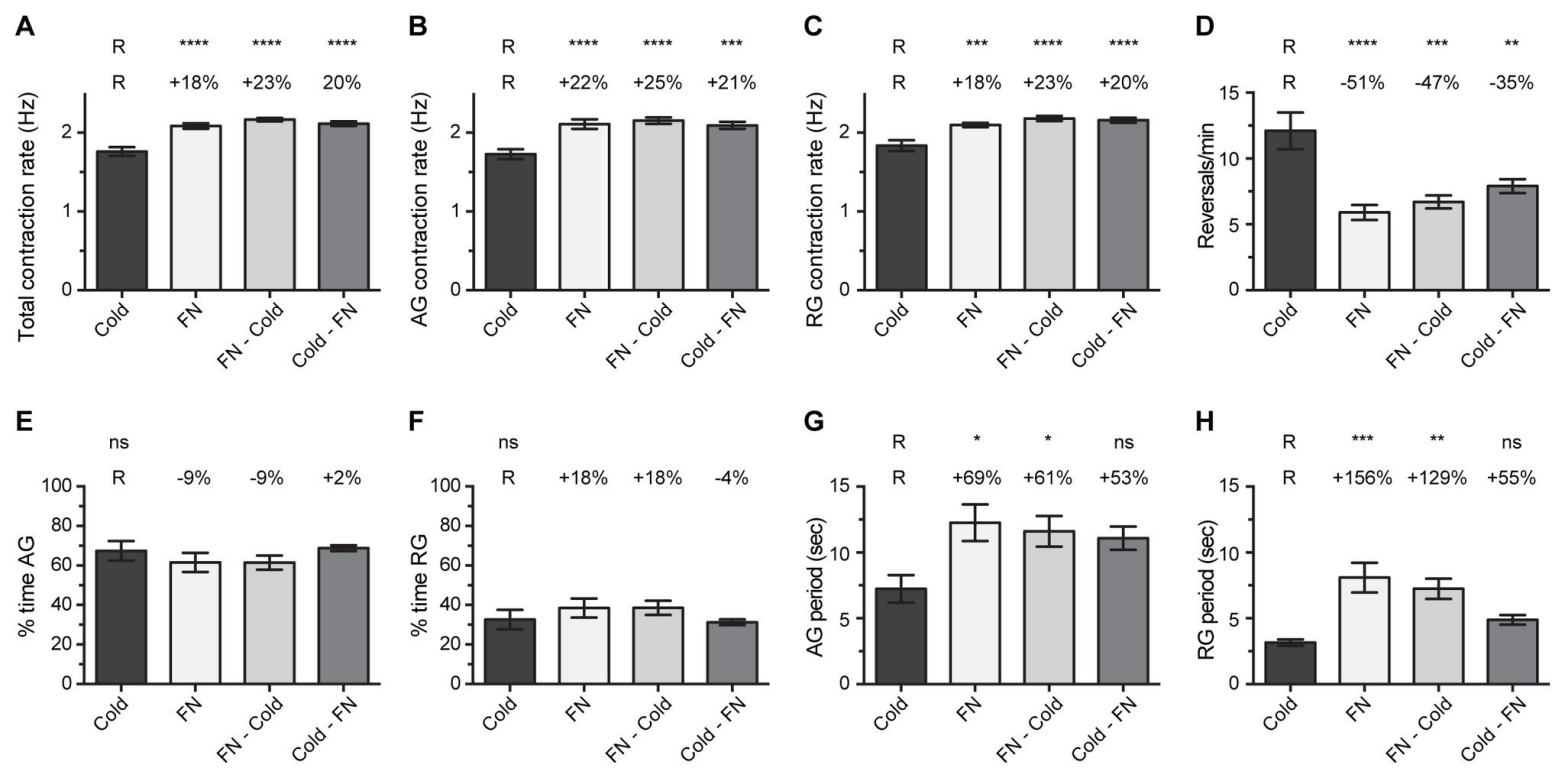
Effect of combining modes of anesthesia on heart physiology. Four groups were assayed: (1) 60 sec cold anesthesia; (2) 30 sec FlyNap (FN) anesthesia; (3) FlyNap anesthesia immediately followed by cold anesthesia; (4) cold anesthesia immediately followed by FlyNap anesthesia. The following heart parameters are reported: (A–C) total, anterograde and retrograde contraction rate; (D) frequency of heartbeat directional reversals; (E–F) percent time contracting in the anterograde and retrograde directions; (G–H) anterograde and retrograde period lengths. The data were analyzed by one-way ANOVA, and when P<0.05 the statistical significances between the cold anesthesia group (R, reference group) and the other groups are denoted using asterisks (Tukey’s multiple comparisons post-hoc test: ns, P>0.05; *, P<0.05; **, P<0.01; ***, P<0.001; ****, P<0.0001). The percentages reported above the columns report the percentage difference between that group and the cold (R) group. Bars denote the standard error of the mean. For all groups, N = 10.

The total heart rate of mosquitoes exposed to cold alone was 1.76 Hz (Figure 3A). Exposure to FlyNap alone resulted in heart rates that were 18% higher than those of mosquitoes that were exposed to cold alone. When mosquitoes were exposed to FlyNap and then cold, the heart rates were 23% higher than mosquitoes anesthetized via cold alone, and when mosquitoes were exposed to cold and then FlyNap, heart rates were 20% higher than mosquitoes that had been exposed to cold anesthesia alone. Similar results were obtained when anterograde and retrograde heart rates were analyzed independently (Figure 3B-C).

The heart of cold-anesthetized mosquitoes reversed contraction direction 12 times per minute (Figure 3D). Exposure to FlyNap, FlyNap and then cold, and cold and then FlyNap led to a 51%, 47% and 35% reduction in the frequency of heartbeat directional reversals, respectively. As a consequence, the groups that included FlyNap (alone or in combination) had longer anterograde and retrograde contraction periods relative to cold anesthesia alone (Figure 3G-H). Finally, the percent time the heart spent contracting in the anterograde and retrograde directions did not change significantly between the treatment groups, although the trend reported in Figure 1 was maintained here (Figure 3E-F).

Together, these data show that the differences in heart rates observed between cold and FlyNap anesthetized mosquitoes are due to a FlyNap induced increase in basal heart rates. Likewise, FlyNap induces a decrease in heartbeat directional reversals, leading to an increase in the length of anterograde and retrograde contraction periods.

### Effect of FlyNap on heart physiology: monitoring across time

From a practical perspective, an advantage of using FlyNap over cold-anesthesia is that FlyNap anesthetizes mosquitoes for several hours while cold anesthetizes mosquitoes for only a few short minutes (Figure 1). Here, we set to test whether heart parameters change during the course of FlyNap anesthesia, and this was done by acquiring heart recordings from individual mosquitoes at both 1 min and 1 h following 30 sec of FlyNap exposure.

**Figure 4:**
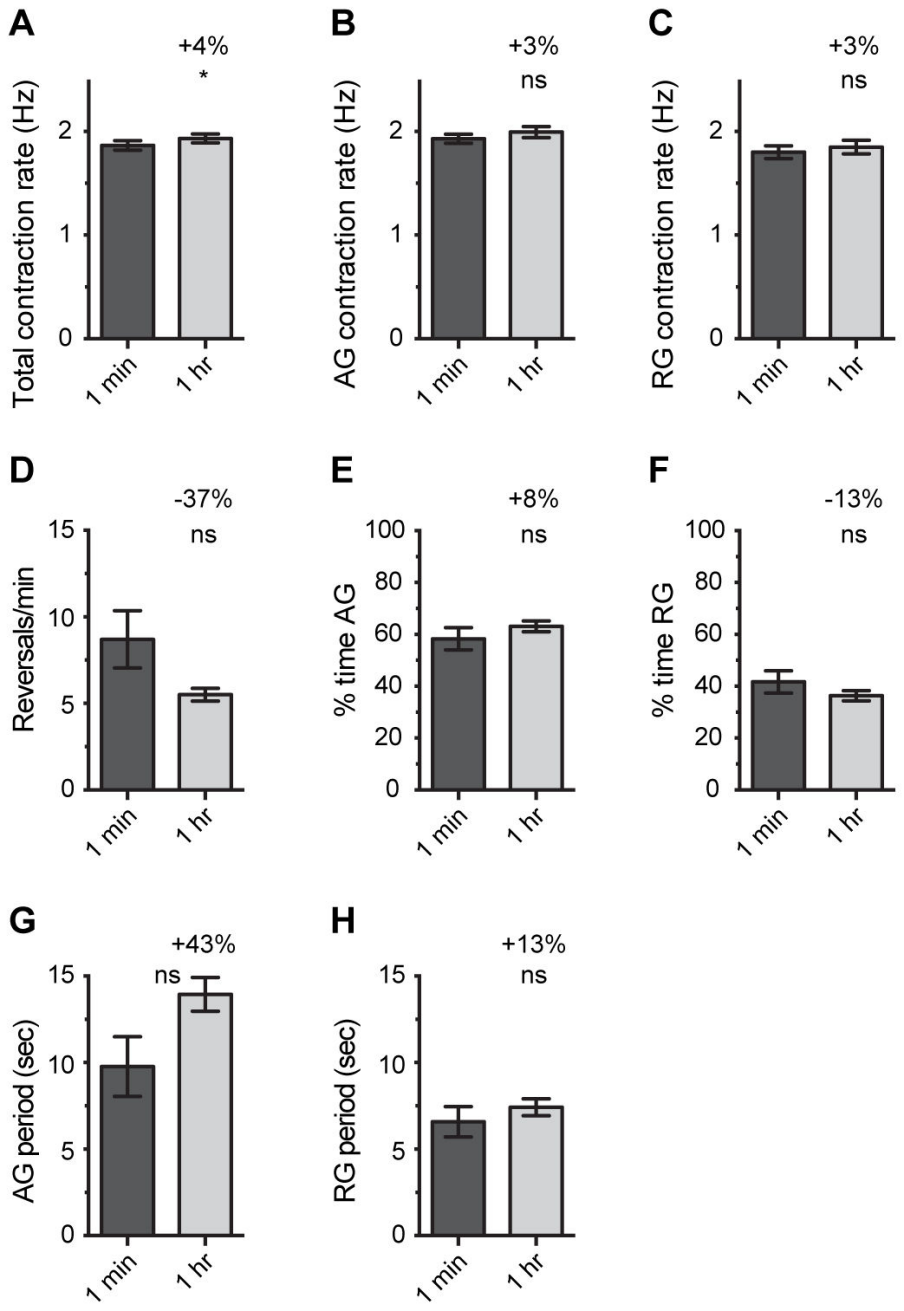
Heart physiology at 1 min and 1 h following FlyNap anesthesia. Mosquitoes were anesthetized by exposure to FlyNap (FN) for 30 sec, and heart recordings were acquired at 1 min and 1 h following FlyNap treatment. The following heart parameters are reported: (A–C) total, anterograde and retrograde contraction rate; (D) frequency of heartbeat directional reversals; (E–F) percent time contracting in the anterograde and retrograde directions; (G–H) anterograde and retrograde period lengths. Data were analyzed using a paired t-test, and the statistical significances between the groups are denoted with an asterisk (ns, P>0.05; *, P=0.0484). The percentages reported in each graph report the percentage change between 1 min and 1 h post-FlyNap treatment. Bars denote the standard error of the mean. N = 10.

The average total heart rate at 1 min post-exposure was 1.87 Hz, and at 1 h post-exposure the average total heart rate was 1.93 Hz (Figure 4A). Although a paired t-test deemed this change to be statistically significant (p=0.0484), the small 3.6% difference between the groups is most likely biologically insignificant. Besides the total heart rate, no other reading was significantly different between the two time points assayed (Figure 4B–H).

### Heart physiology following cold or FlyNap anesthesia: effect of CCAP

We have shown that crustacean cardioactive peptide (CCAP) is a cardioacceleratory neurohormone in mosquitoes [19], and others have shown that CCAP is cardioacceleratory in a broad range of arthropods [30–36]. To determine whether mosquitoes anesthetized by exposure to FlyNap remain responsive to this myotropic neuropeptide, heart recordings were acquired (1) 1-5 min after mosquitoes had been anesthetized via either exposure to cold or via exposure to FlyNap for 30 sec, and (2) 10 min after those same mosquitoes had been injected with either PBS or 1 X 10^-5^ M CCAP in PBS (diluted in the hemolymph to ~1-2 X10^-6^ M). The dose of 1 X 10^-5^ M CCAP was selected for this experiment as in a previous study we showed that this treatment increases the heart contraction rate by 24% [19].

**Figure 5:**
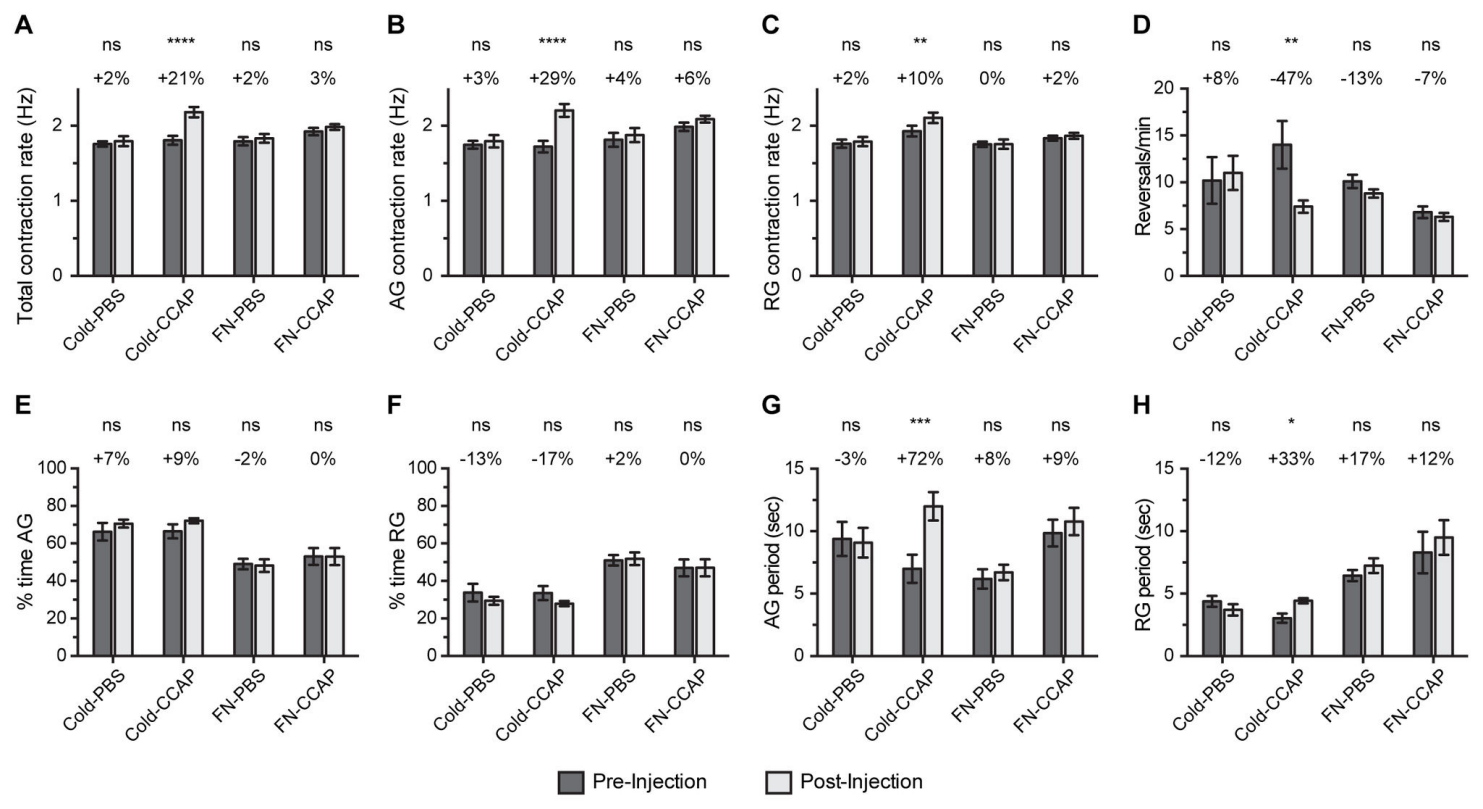
Effect of CCAP on heart physiology following cold or FlyNap anesthesia. Four groups were assayed: (1) cold anesthesia followed by PBS injection; (2) cold anesthesia followed by 1 X 10^-5^ M CCAP in PBS; (3) 30 sec FlyNap (FN) anesthesia followed by PBS injection; (4) 30 sec FlyNap anesthesia followed by 1 X 10^-5^ M CCAP in PBS. The following heart parameters are reported: (A–C) total, anterograde and retrograde contraction rate; (D) frequency of heartbeat directional reversals; (E–F) percent time contracting in the anterograde and retrograde directions; (G–H) anterograde and retrograde period lengths. Data were initially analyzed by repeated measures two-way ANOVA, and for each treatment group the statistical significances between the pre- and post-injection datasets are denoted using asterisks (Sidak’s multiple comparison’s post-hoc test: ns, P>0.05; *, P<0.05; **, P<0.01; ***, P<0.001; ****, P<0.0001). The percentages reported above each set of columns report the percentage change between pre- and post-injection. Bars denote the standard error of the mean. For all groups, N = 10.

Similar to what we have previously reported [19], injecting PBS into cold anesthetized mosquitoes led to a negligible (2%) increase in the total heart rate while CCAP injection led to a significant, 21% increase in the total heart rate (Figure 5A). PBS injection did not significantly affect the heart rate in either direction, but CCAP injection led to a much larger increase in the anterograde contraction rate (29%) than in the retrograde contraction rate (10%; Figure 5B-C). In mosquitoes anesthetized by cold exposure, CCAP treatment also led to a decrease in the frequency of heartbeat directional reversals, which resulted in significantly longer contraction periods in both the anterograde and retrograde directions (Figure 5D, G-H).

Although CCAP treatment had a profound effect on cardiac physiology in cold anesthetized mosquitoes, FlyNap treatment rendered the heart insensitive to CCAP. Similar to cold-anesthetized mosquitoes, injecting PBS into FlyNap anesthetized mosquitoes led to negligible 2%, 4% and 0% increases in the total, anterograde and retrograde contraction rates, respectively (Figure 5A–C). However, unlike that seen in cold anesthetized mosquitoes, injecting CCAP into FlyNap anesthetized mosquitoes led to negligible (non-significant) changes in the contraction rate: neuropeptide injection increased the total, anterograde and retrograde contraction rates by only 3%, 6% and 2%, respectively (Figure 5A–C). Finally, CCAP treatment of FlyNap anesthetized mosquitoes did not affect any of the other heart parameters measured (Figure 5D–H). In summary, these data show that the cardio-modulatory function of CCAP is lost when mosquitoes are exposed to FlyNap.

### Effect of cold versus FlyNap anesthesia on immune competence

**Figure 6:**
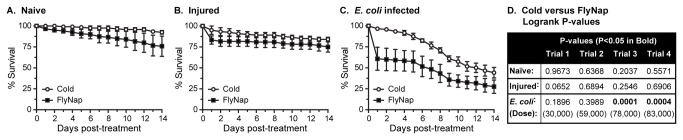
Effect of cold or FlyNap anesthesia on immune competence. Mosquitoes were anesthetized by exposure to cold or by exposure to FlyNap for 30 sec. A-C. Survival curves of naïve (A), injured (B) and *E. coli* infected (C) mosquitoes. The curves presented are the average of four trials composed of 30 mosquitoes per treatment group, and bars denote the standard error of the mean. D. LogRank P values comparing the cold and FlyNap survival curves for the treatment groups in all 4 independent trials.

To determine whether FlyNap affects a mosquito’s ability to overcome a bacterial infection we tracked mosquito survival following cold anesthesia and following 30 sec FlyNap anesthesia in naïve (not manipulated), injured (injected with LB broth) and infected (injected with *E. coli* in LB broth; infection dose ranged from 30,000 to 83,000 bacteria per mosquito) individuals. By 14 days post-treatment, 93% and 76% of naïve mosquitoes that had been exposed to cold or FlyNap remained alive, respectively (Figure 6A). Relatively similar values were recorded for injured mosquitoes: by 14 days post-treatment, 84% and 75% of injured mosquitoes that had been exposed to cold or FlyNap remained alive, respectively (Figure 6B). Logrank statistical analysis comparing the survival curves of naïve or injured mosquitoes did not detect a significant difference between the two modes of anesthesia (Figure 6D). Interestingly, the majority of injured mosquitoes that died following FlyNap exposure did so during the first 24 h post-treatment (83% survival; slope = -16.667). After the first 24 h following injury, the survival slopes of cold exposed (slope = -0.8168; R^2^ = 0.9697) and FlyNap exposed (slope = -0.5238; R^2^ = 0.9014) mosquitoes were relatively similar.

The mode of anesthesia had a significant effect on a mosquito’s ability to survive a bacterial infection (Figure 6C-D). By 14 days post-infection, 44% of the mosquitoes anesthetized via exposure to cold remained alive, but only 28% of the mosquitoes exposed to FlyNap had survived. The form of anesthesia had a statistically significant effect on the survival curves in two of the four trials, and interestingly, the two trials that were statistically different were those with the highest infection doses (Figure 6D). However, similar to what was observed following injury, most infected mosquitoes in the FlyNap treated groups perished within the first 24 h post-treatment (61% survival; slope = -39.167). After the first 24 h following infection, the survival slopes of cold exposed (slope = -4.6465; R^2^ = 0.9745) and FlyNap exposed (slope = -3.011; R^2^ = 0.9544) mosquitoes were relatively similar.

## Discussion

FlyNap, which contains the active ingredient triethylamine, is routinely used to anesthetize *Drosophila* fruit flies [13–16]. Lethal doses of triethylamine have been used to permanently immobilize mosquitoes in both laboratory and field studies [6–11], but the use of FlyNap or triethylamine to temporarily anesthetize mosquitoes has not been reported. Thus, in search of a suitable alternative to cold anesthesia (the most widely used method to immobilize anophelines), we set to determine whether FlyNap is a suitable anesthetic agent for research into mosquito immunology and circulatory physiology.

Comparison of heart contraction dynamics in FlyNap anesthetized mosquitoes versus mosquitoes anesthetized using the traditional cold procedure showed that exposure to FlyNap doses that are ultimately lethal (the mosquito dies several hours following treatment) lead to a decrease in mosquito heart rates, presumably because of the terminal disruption of physiological processes. However, non-lethal doses of triethylamine leads to an increase in the heart rate, a decrease in the frequency of heartbeat directional reversals, and an increase in the percentage of the time the heart spends contracting in the retrograde direction. Experiments employing various combinations of cold and FlyNap anesthesia showed that cold anesthesia does not affect basal heart physiology, and that the differences seen between the cold and the FlyNap groups are due to a FlyNap-induced alteration of heart physiology. Hence, these data suggest that FlyNap affects mosquito heart physiology.

In *Drosophila melanogaster* several studies have assessed cardiac physiology following FlyNap-based anesthesia [16,37–41]. Of these studies, two compared FlyNap anesthesia to other methods of restraint, with the authors of these studies reaching different conclusions. First, Pasternostro et al. [16] concluded that, in comparison to CO_2_ and ether anesthesia, FlyNap causes the least amount of cardiac disruption. This conclusion was based on the finding that CO_2_ causes temporary cardiac arrest and ether depresses heart rates. Then, Tsai et al. [40] showed that FlyNap and CO_2_ anesthesia induce irregular heart rhythms in fruit flies when compared to animals that had been attached to a substrate using an epoxy resin. In addition, they showed that FlyNap elevates heart rates relative to epoxy-based restraint. While the conclusions in these two studies are seemingly opposite, they are the product of the comparisons being made. CO_2_ anesthetizes insects by depriving them of oxygen, so it is not surprising that this causes cardiac arrest. Thus, comparing CO_2_ to FlyNap would result in the conclusion that FlyNap is the better anesthetic agent. Epoxy-based anesthesia, on the other hand, simply adheres the fly to a substrate. While this could bias circulatory physiology by restricting the abdominal contractions known to facilitate extracardiac hemolymph propulsion [17,42], this method, assuming that the spiracles are not blocked, does not affect respiration or any other physiological process. Thus, comparing resin to FlyNap would result in the conclusion that epoxy is the better anesthetic agent. Furthermore, although it is difficult to make horizontal comparisons across studies, it is interesting that the heart rates of FlyNap anesthetized flies in the Pasternostro et al. [16] and Tsai et al. [40] studies are higher than the reported *Drosophila* heart rates in studies where the flies were immobilized using either cold or an adhesive [31,43,44]. Together, these reports are consistent with our finding that FlyNap induces an increase in the mosquito heart rate.

In addition to increasing heart rates, FlyNap has a negative effect on a mosquito’s ability to survive a bacterial infection. Interestingly, the majority of FlyNap associated mortality was observed during the first day after injury or infection, and mosquitoes that survived the first day post-infection (or injury) survived equally as well as their cold anesthesia counterparts. The reason for this FlyNap associated mortality is not clear, but it is possible that a small amount of this chemical is introduced into the hemocoel during the process of injection, which weakens the mosquito and reduces its ability to seal wounds and fight off invaders.

The mechanism by which FlyNap affects mosquito heart physiology is not clear. Not much is known about the molecular effects of triethylamine on organ systems but this chemical has been shown to stimulate the vertebrate central nervous system by inhibiting monoamine oxidase [45,46]. Monoamine oxidases break down monoaminergic neurotransmitters such as serotonin and dopamine [47]. Given that elevated levels of either of these neurotransmitters lead to increased *Drosophila* heart rates [48], it is possible that triethylamine-induced increases in hemocoelic serotonin and dopamine levels lead to elevated mosquito heart rates. However, another explanation for the elevated heart rates observed following FlyNap exposure might be more likely. Triethylamine is a paralytic agent that also eliminates the cardiac response to CCAP. Thus, we hypothesize that this chemical, among other things, disrupts the effect of endocrine signaling, thus eliminating the neurohormonal component of heart modulation and motor movement in general. In the case of CCAP, this effect must be at the level of the receptor or downstream signaling, as introduction of the neurohormone into FlyNap treated mosquitoes does not affect heart physiology. Moreover, the hypothesis that triethylamine disrupts endocrine signaling could also explain the negative effect that FlyNap has on mosquito survival during the first 24 h of infection, as insulin signaling and other endocrine factors modulate mosquito immune responses [49,50].

In light of our hypothesis that triethylamine disrupts endocrine signaling, these data support the currently held belief that, although the insect heart contracts in a myogenic manner, its rhythmicity and directionality is modulated by neurohormonal stimuli [51–53]. Specifically, our finding that triethylamine does not impede heart contractions supports the myogenic nature of the heart, and is in agreement with studies showing that neuromuscular paralysis or the separation of the heart from the underlying neural tissue does not abolish heart contractions [54,55]. Moreover, the neurohormone CCAP elevates heart rates in diverse insect orders [19,30–32,34,36], showing that neurohormones are involved in regulating heart rhythmicity. In *D. melanogaster* CCAP has also been shown to function as an anterograde pacemaker [31], and this trend was also observed in *A. gambiae* [19]. Three independent datasets in this study jointly support the cardioacceleratory function of CCAP and its role as an anterograde pacemaker. First and most obvious, FlyNap treatment eliminates the cardioacceleratory activity of CCAP. Second, when compared to cold anesthesia, FlyNap treatment reduces the percent time that the heart spends contracting in the anterograde direction, bringing the anterograde–retrograde ratio close to 1:1 instead of the typical 2:1 that is observed following cold anesthesia. Third, following cold anesthesia CCAP has a more pronounced effect on the anterograde heart rate than in the retrograde heart rate. Together, these data support the hypothesis that the myogenic heart is under partial neurohormonal control, and that CCAP functions as an anterograde pacemaker.

## Conclusions

In summary, the initial goal of this study was to determine whether FlyNap is a suitable anesthetic agent for experiments assessing mosquito immunology and circulatory physiology. We conclude that FlyNap is not an appropriate anesthetic agent for studies assessing heart function. Although for practical reasons FlyNap may still be useful for studies where prolonged immobilization is required or for studies where the restraint system interferes with cardiac recordings, we recommend cold anesthesia for experiments assessing heart function. Mainly (1), immobilization with cold does not interfere with respiration or abdominal contractions (2), the insect’s temperature normalizes fairly quickly following a return to ambient temperature, and (3) the heart remains sensitive to neurohormonal input. Moreover, the data generated in this study also illustrate the intricate biology of the insect heart. Specifically, that the heart is under neurohormonal control and that CCAP serves as an anterograde pacemaker.
